# HIV sero-status disclosure and associated factors among HIV positive women in East Africa: Systematic review and meta-analysis. Implications for prevention of mother-to-child HIV transmission

**DOI:** 10.3389/fpubh.2022.919410

**Published:** 2022-11-22

**Authors:** Getu Mosisa, Diriba Mulisa, Adugna Oluma, Lami Bayisa, Emiru Merdassa, Diriba Bayisa, Afework Tamiru, Tadesse Tolossa, Dereje Chala Diriba, Getahun Fetensa, Bizuneh Wakuma

**Affiliations:** ^1^School of Nursing and Midwifery, Institutes of Health Sciences, Wollega University, Nekemte, Ethiopia; ^2^Department of Public Health, Institutes of Health Sciences, Wollega University, Nekemte, Ethiopia; ^3^Deakin Health Economics, School of Health and Social Development, Institute for Health Transformation, Deakin University, Geelong, VIC, Australia

**Keywords:** disclosure status, HIV, women, East Africa, meta-analysis

## Abstract

**Background:**

Women's HIV-positive disclosure plays a pivotal role to achieve the goal of preventing mother-to-child transmission (PMTCT) among pregnant women in particular. Although several primary studies were conducted in the different countries of East Africa, no study concluded the prevalence of women's HIV status disclosure and associated factors in East Africa. Therefore, the current study aimed to assess the pooled prevalence of disclosure status and associated factors among women in East Africa.

**Objectives:**

To assess the pooled prevalence of HIV sero-status disclosure and associated factors among women in East Africa.

**Methods:**

HINARI, PubMed, and Cochrane Library databases were searched. The data were extracted using a Microsoft Excel spreadsheet and STATA v 14.1 was used for the analysis. The Funnel plots and Egger's statistical test was used to check publication bias. Heterogeneity was assessed by conducting sensitivity and subgroup analyses.

**Result:**

The pooled prevalence of sero-status disclosure among women in East Africa was 73.77% (95%CI 67.76, 79.77). Knowing partner's sero-status (OR = 10.04(95%CI 3.36, 31.84), married (OR = 2.46 (95%CI 1.23, 4.89), smooth relationship (OR = 3.30 (95%CI 1.39, 7.84), and discussion on HIV before the test (OR = 6.96 (95%CI 3.21, 15.05) were identified determinants of HIV sero-status disclosure.

**Conclusion:**

The current systematic and meta-analysis revealed that nearly one-fourth of women had not disclosed HIV sero-status to at least one individual. Knowing the partner's HIV sero-status, being married, having a smooth relationship, and discussing on HIV before the test were determinants of disclosure status. Therefore, disclosure of HIV-positive sero-status among women living with HIV needs to be strengthened.

## Introduction

Human Immunodeficiency virus (HIV) continues to be one of the health and development challenges globally. By 2020, about 37.7 million people were living with HIV/AIDS (1). Eastern and Southern Africa is the epicenter of the HIV burden; they contributed nearly 20.7 million HIV/AIDS clients to a global total (2).

In addition to lifelong therapy, the role of sero-status disclosure to reduce onward HIV transmission is witnessed in literature particularly in preventing mother-to-child transmission (PMTCT) (3). Disclosure is the process of enlightening HIV-positive status to a sexual partner, family members, and in one's social networks over time (4). It is a personal and intimate process that engages the soul, the mind, and the body that shapes the self-image, self-efficacy, self-perception, and confidence of HIV-infected individuals (5).

Disclosing HIV seropositive status has an imminent role in both prevention and management (6); smoothing social support, increasing adherence, expanding awareness, and reducing risky behaviors (7).

Studies exposed that HIV-positive disclosure among pregnant women is attributed to different factors including residence (8), educational level (9), low- income (10), Fear of stigma (11), and disturbed relationships (12–15). In other literature, disclosure status is explained by adherence to antiretroviral therapy (ART), confidentiality problems, and the provider's expressed anxiety (16–18). Additionally, social context can be a key dimension of disclosure as an impression for financial, social support, and emotional support, and help with medical care or counseling; disclosure is higher when hoping to receive help and lower when expecting blame and discrimination (19, 20).

Therefore, this systematic review and meta-analysis aimed to determine the pooled prevalence and associated factors of disclosure status among HIV-positive Women in East Africa. The finding will enable scholars, clinicians, and policymakers to appraise the existing approach and create a new HIV prevention and control strategy for PMTCT.

## Methods

### Search strategies

**Figure 1:**
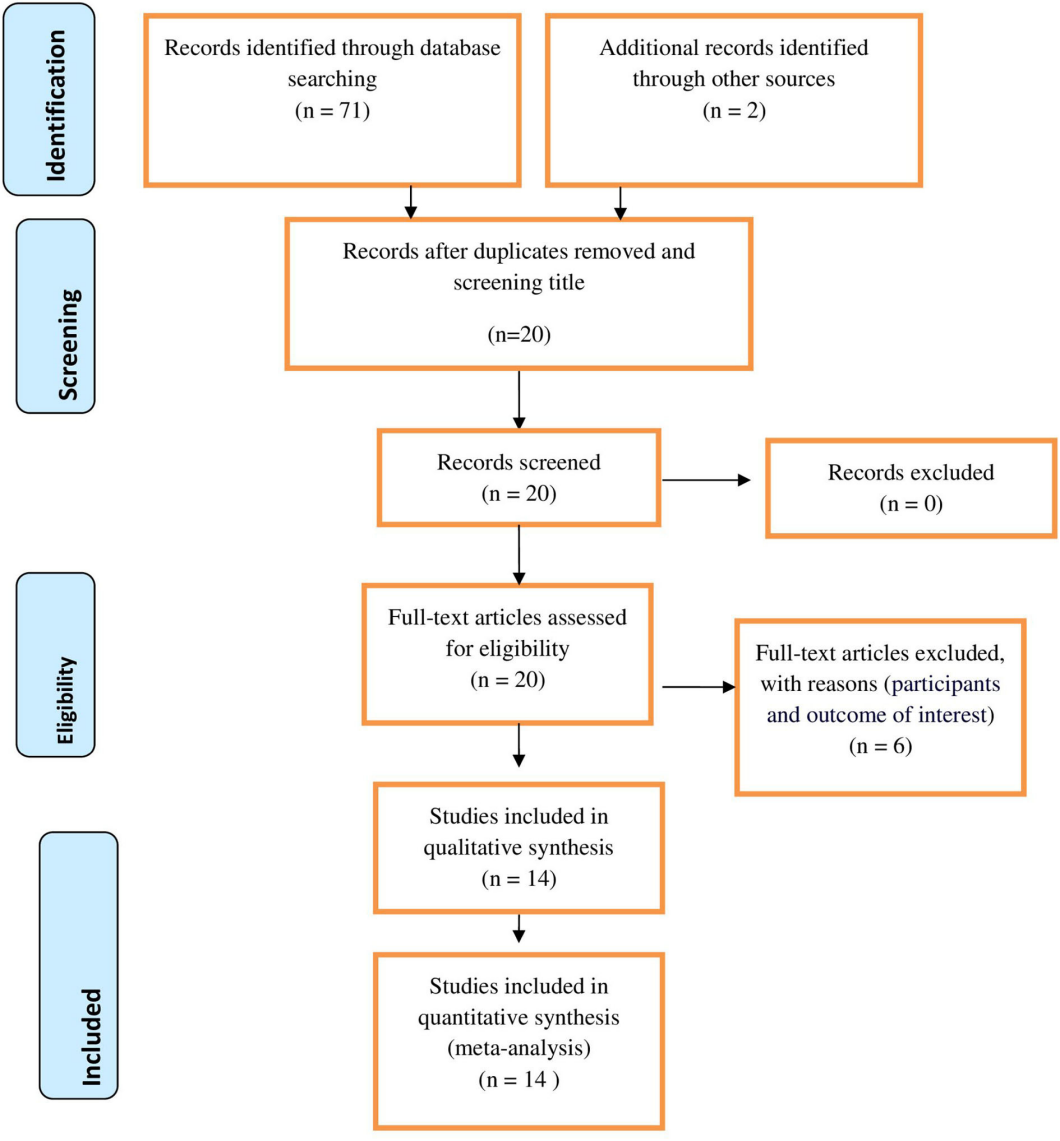
PRISMA flow diagram of included studies for the systematic review and meta-analysis of disclosure status and associated factors among Women in East Africa, 2021.

PubMed, HINARI, and Cochrane Library databases were searched. The literature search was performed from March 1 to March 30, 2021. Searches were conducted using terms such as “HIV sero-status disclosure,” “disclosure status,” “magnitude,” “prevalence,” “associated factors,” “determinants” “Pregnant women,” “Lactating women,” Women,” HIV positive,” and “lists of all East African countries.” Boolean operators like “AND” and “OR” were used. PRISMA checklist and flow diagram were used for reporting the procedure (Figure 1).

### Selection and eligibility criteria

Studies that were conducted on the disclosure status among women living with HIV in East Africa were included. Each article was independently reviewed by two investigators (GM & DM). The Study populations were all WLHIV in East Africa. Studies conducted in East Africa were included in the review. All observational studies reporting disclosure status among WLHIV in East Africa were included. Articles reported in the English language were included. Articles that were not fully accessible, after at least two email contact attempts with the primary authors, were excluded.

### Outcome measurement

Disclosure status among WLHIV was the primary outcome of this study. It was categorized as “yes” if the HIV-positive women disclosed their status to at least one individual including sexual partner, family, husband, and relatives, and “no” if not disclosed to a least one of them. It is measured as the total number of disclosed cases over a total number of all women multiplied by 100. The second outcome was factors associated with disclosure status among WLHIV. For the second outcome, the log odds ratio was determined to see the association between disclosure status and associated factors. Factors included in this review include knowing the partners sero-status (know vs. not know), educational status (no education/primary vs. secondary and above), the relationship before the test (smooth vs. disagreement), marital status (married vs. unmarried), residence (rural vs. urban), and discussion on HIV before the test (absence vs. presence).

### Data extraction and quality assessment

Joanna Briggs Institute Critical Appraisal Checklist for Analytical Cross-Sectional Studies was used to assess the quality of included studies (21). The data were extracted by two authors (GM and DM) using data extraction checklists on Microsoft excel. Reference management software (Endnote version X7.2) was used to combine search results from databases and to remove duplications. Studies were screened using abstracts and titles. Then, the eligibility of the studies was evaluated using predetermined inclusion and exclusion criteria. For the outcome, disclosure status, and associated factors, data were extracted in the format of two-by-two tables. The log odds ratio was calculated based on the findings of the primary studies. The data extraction checklist contains the author's name, country, study design, year of publication, sample size, response rate, and the number of participants with the outcome. Any disagreement between two independent reviewers was resolved by involving a third reviewer (AO).

### Statistical analysis

Data were extracted from original articles and then exported to STATA v 14 for analysis. The prevalence of disclosure status with a 95% confidence interval (CI) and OR of the associated factors was presented in the form of a forest plot. Cochran *Q* test (chi-squared statistic) and I^2^ statistic on forest plots were used to check heterogeneity among the included studies. Sensitivity and subgroup analyses were conducted to assess the presence of heterogeneity among primary studies. A country-based sub-group analysis was performed as heterogeneity was observed. As heterogeneity was observed for the first outcome, a random-effects model was used to determine the prevalence of disclosure status. To test publication bias, a funnel plot and Egger's weighted regression tests were computed.

## Results

### Study selection

Seventy-one articles were identified through different database searches. Of those, 51 articles were removed due to duplications. Then, 6 articles were removed because they failed to meet the eligibility criteria. Fourteen articles that have a score of seven and above on the JBI quality appraisal were included in the analysis. PRISMA flow diagram was used to present the selection process.

### Characteristics of included studies

About 4,365 study populations were involved in this review. The sample size ranges from a minimum of 42 to a maximum of 665 from Ethiopia. Of all studies, eight (22–29) were from Ethiopia, three (30–32) were from Uganda and three (33–35) were from Tanzania. All included articles were cross-sectional studies (Table 1).

**Table 1:** Summary of included studies on magnitude of HIV sero-status disclosure among women in East Africa, 2021.

**References**	**Year of publication**	**Country**	**Study design**	**Sample size**	**Prevalence (95%CI)**
Meseret et al. (22)	2019	Ethiopia	Cross-sectional	665	80.60 (77.60, 83.61)
Sendo et al. (23)	2013	Ethiopia	Cross-sectional	112	69.64 (61.13, 78.16)
Kassaye et al. (24)	2005	Ethiopia	Cross-sectional	42	69.05 (55.07, 83.03)
Kassahun et al. (25)	2018	Ethiopia	Cross-sectional	337	86.05 (82.35, 89.75)
Damian et al. (35)	2019	Tanzania	Cross-sectional	609	65.68 (61.91, 69.45)
Alemayehu et al. (26)	2014	Ethiopia	Cross-sectional	315	63.81 (58.50, 69.12)
Kiula et al. (33)	2013	Tanzania	Cross-sectional	250	40.80 (34.71, 46.89)
Alemayehu et al. (27)	2014	Ethiopia	Cross-sectional	263	89.73 (86.07, 93.40)
Deribe et al. (28)	2018	Ethiopia	Cross-sectional	207	72.95 (66.90, 79.00)
Ngonzi et al. (30)	2019	Uganda	Cross-sectional	103	85.44 (78.62, 92.25)
Oshosen et al. (34)	2016	Tanzania	Cross-sectional	167	74.85 (68.27, 81.43)
Batte et al. (31)	2015	Uganda	Cross-sectional	408	83.82 (80.25, 87.40)
Tolossa et al. (29)	2021	Ethiopia	Cross-sectional	380	73.42 (68.98, 77.86)
Naigino et al. (32)	2017	Uganda	Cross-sectional	507	73.96 (70.14, 77.78)
Overall with weights from random effect					73.77 (67.76, 79.77)

### Prevalence of HIV sero-status disclosure among WLHIV

**Figure 2:**
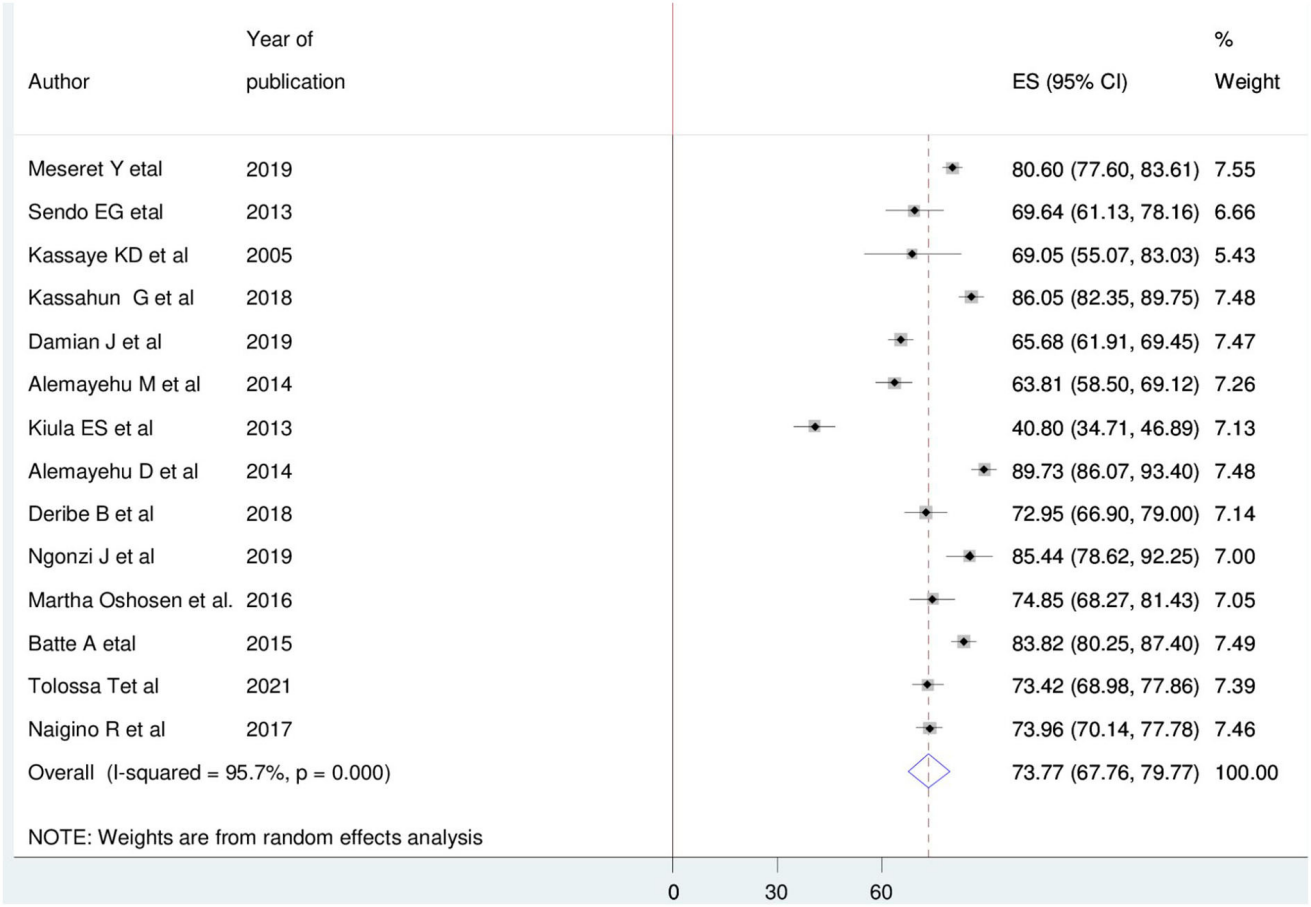
Forest plot of the pooled magnitude of HIV positive sero-status disclosure among women in East Africa, 2021.

The pooled prevalence of HIV-positive disclosure status among women in East Africa was 73.77% (95% CI: 67.76, 79.77). The largest prevalence was observed from Ethiopia 89.73% (95% CI: 86.07, 93.40) (27), while the smallest was from Tanzania 40.80% (95% CI: 34.71, 46.89) (33). Due to heterogeneity, the magnitude of HIV-positive disclosure among women was computed using a random-effect model (I^2^= 95.7%, *P* = < 0.001) (Figure 2).

### Factors related with heterogeneity

High heterogeneity was observed (I-squared = 95.7%, *p* = < 0.001). However, the source of heterogeneity is not due to sample size and year of publication (Table 2).

**Table 2:** Factors related with heterogeneity of HIV-positive sero-status disclosure among women in East Africa, 2021.

	**Coefficient**	***P*-value**	**95%CI**	
Year of publication	1.217492	0.298	−1.235329	3.670313
Sample size	−0.0090618	0.696	−0.0587687	0.0406451

### Subgroup analysis and publication bias

**Figure 3:**
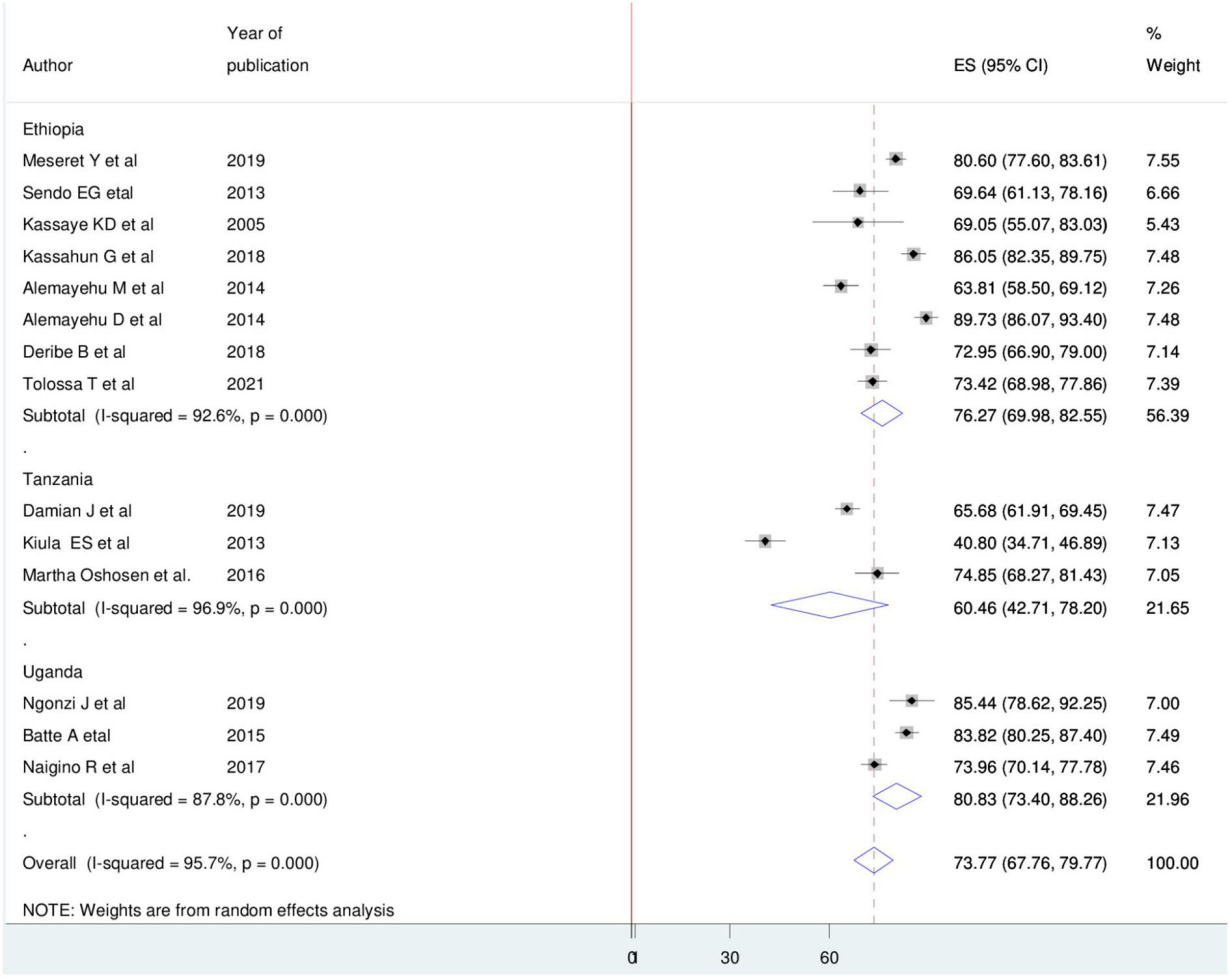
Subgroup analysis on pooled magnitude of HIV positive sero-status disclosure among women in East Africa, 2021.

**Figure 4:**
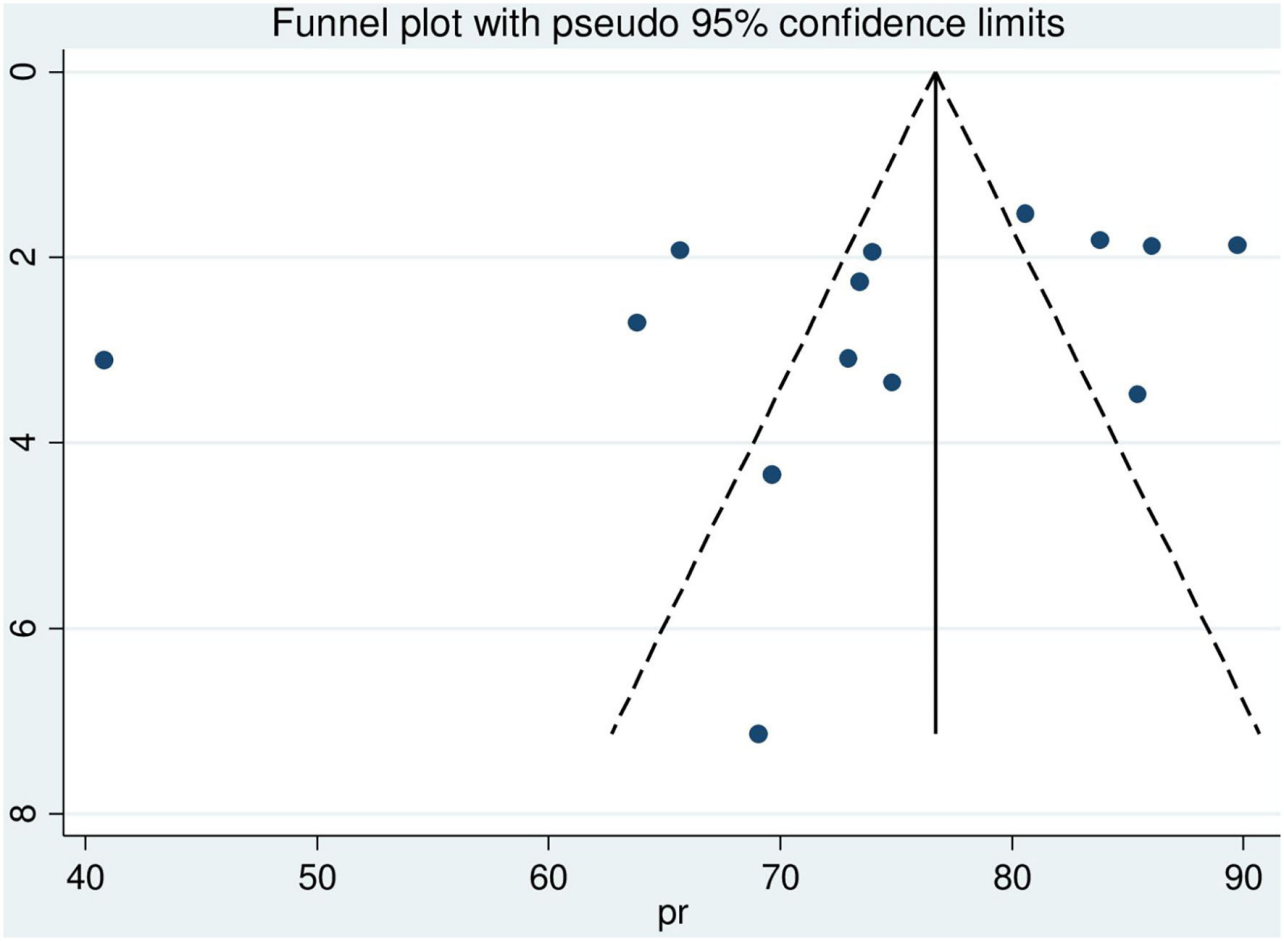
Funnel plot with 95% confidence limit of HIV sero-status disclosure women in East Africa, 2021.

Country-based subgroup analysis was conducted. Accordingly, the prevalence ranged from the smallest in Tanzania 60.46% (95% CI: 42.71, 78.20) (33–35) to the largest in Uganda 80.83% (95% CI: 73.40, 88.26) (30–32) (Figure 3). The funnel plot and Egger test were computed to assess the publication bias. As a result, the funnel plot shows a shape of symmetry (Figure 4), and the Egger test showed no statistical significance for the presence of publication bias (*p*-value = 0.16).

### Sensitivity analysis

**Figure 5:**
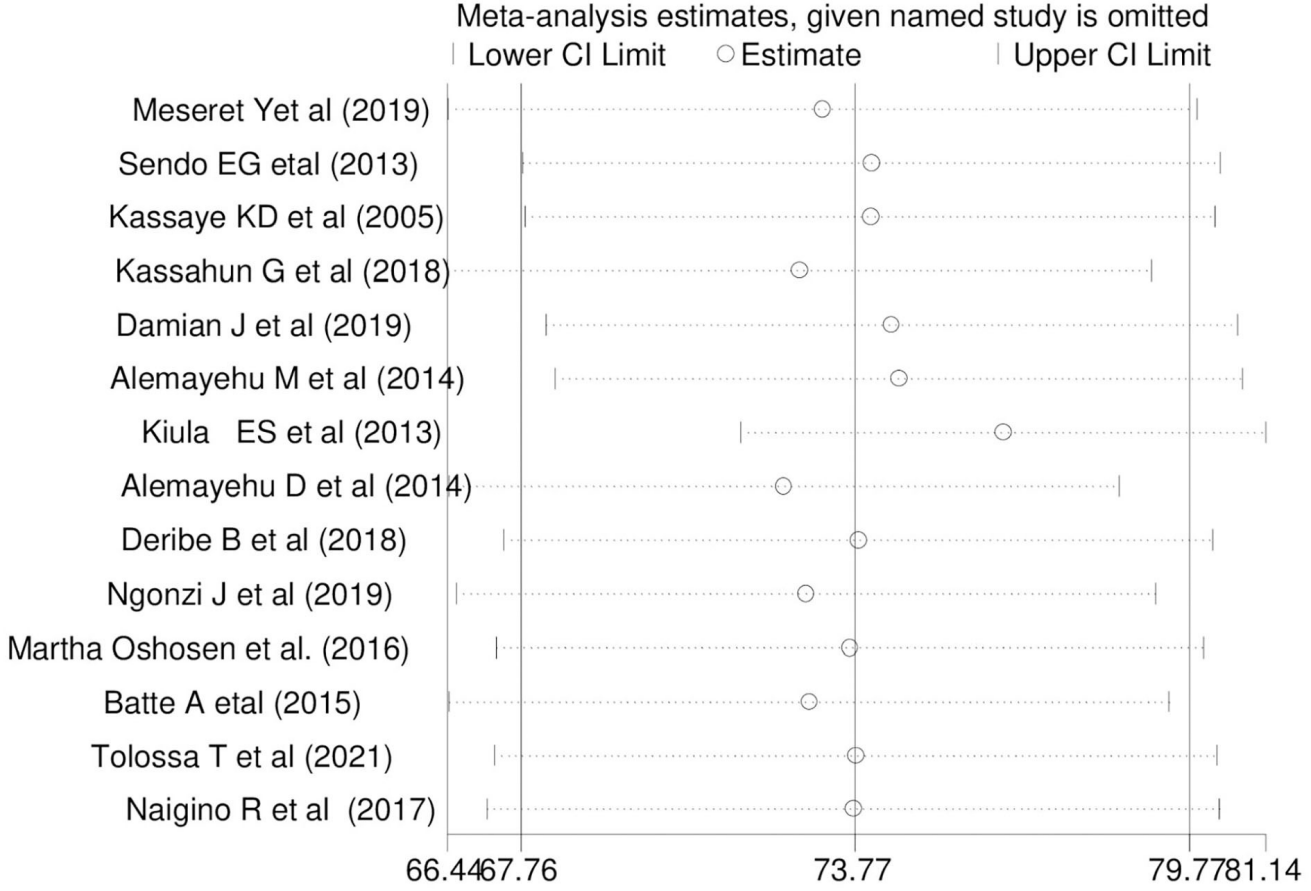
Sensitivity analysis for single study influence of the pooled magnitude of HIV sero-status disclosure women in East Africa, 2021.

In this review, to identify the influence of a single study on the overall meta-analysis, sensitivity analysis was performed using a random-effects model. The result showed there was no strong evidence for the effect of a single study's influence on the overall meta-analysis (Figure 5).

### Factors associated with HIV sero-status disclosure among women

#### Association between HIV sero-status disclosure and knowing partner HIV status

**Figure 6:**
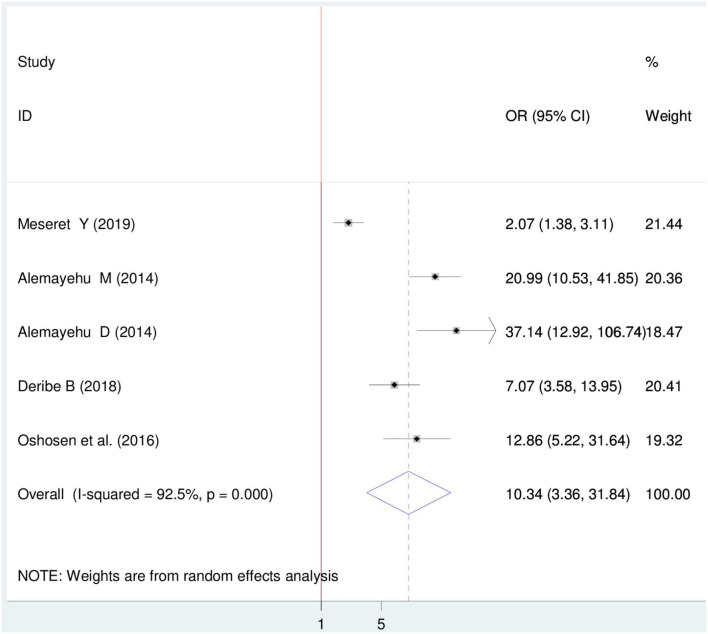
Forest plot of association between HIV sero-status disclosure and knowing partner HIV status among women in East Africa, 2021.

Five articles were included (22, 26–28, 34) and the pooled finding indicated that there is a significant association between HIV sero-status disclosure and knowing the partner's HIV status. The result indicated that women who know their partner's HIV status were 10.04 times more likely to disclose HIV sero-status than those who do not know their partner's status OR = 10.04(95%CI 3.36, 31.84) Cite. In this finding, a random-effect model was used (I^2^ = 92.5%, *P* = < 0.001) (Figure 6).

#### Association between HIV sero-status disclosure and marital status

**Figure 7:**
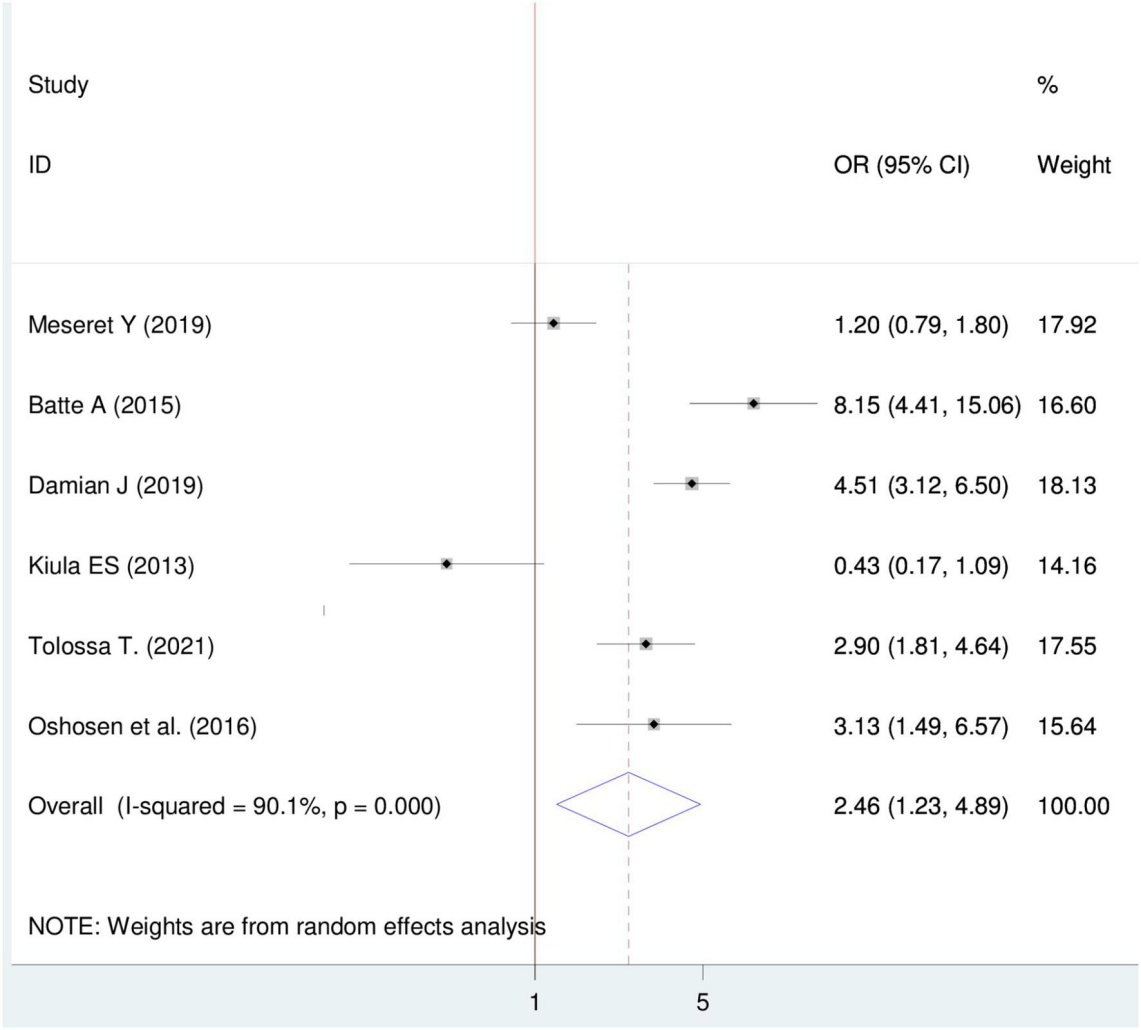
Forest plot of association between HIV sero-status disclosure and marital status, among women in East Africa 2021.

Six articles were included (22, 29, 31, 33–35). Four articles indicated a positive association (29, 31, 34, 35) while one article showed a negative association with disclosure status. The pooled finding showed a significant association between HIV sero-status disclosure and marital status. Thus, married women were 2.46 times more likely to disclose HIV sero-status than those who are single OR = 2.46 (95%CI 1.23, 4.89). In this finding, a random effect model was used (I^2^ = 90.01%, *P* = < 0.001) (Figure 7).

#### Association between HIV sero-status disclosure and relationship before the test

**Figure 8:**
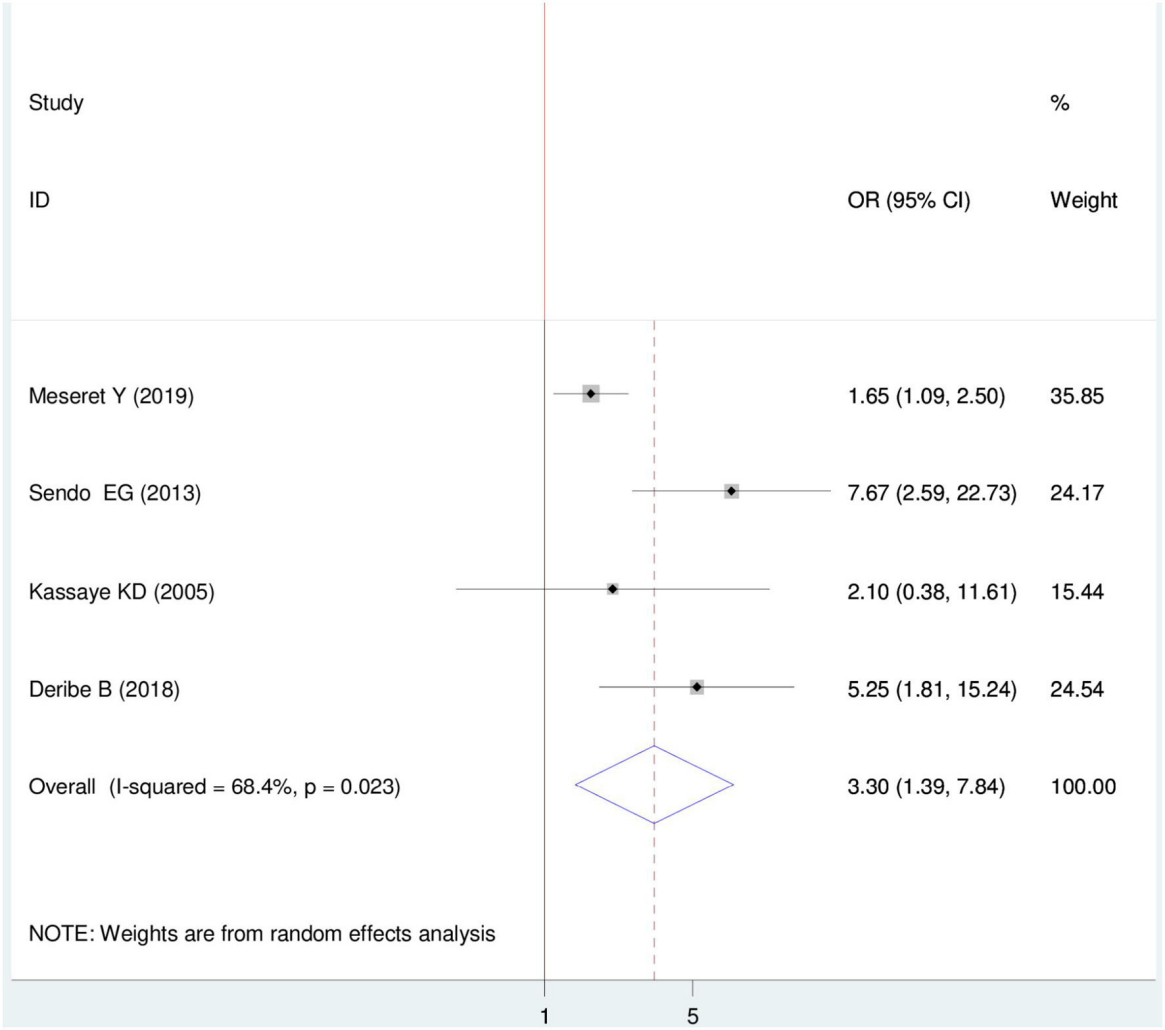
Forest plot of association between HIV sero-status disclosure and relationship before test among women in East Africa, 2021.

Four articles were included (22–24, 28) in which three articles showed a significant association between HIV sero-status disclosure and the relationship before the test. The Meta-regression finding showed that there is a significant association between HIV sero-status disclosure and a relationship before the test. Though, women who have a smooth relationship were 3.30 times more likely to disclose HIV sero-status than those who have disagreements with their partner OR = 3.30 (95%CI 1.39, 7.84). In this finding, a random effect model was used (I^2^ = 68.04%, *P* = 0.023) (Figure 8).

#### Association between HIV sero-status disclosure and discussion before the test

**Figure 9:**
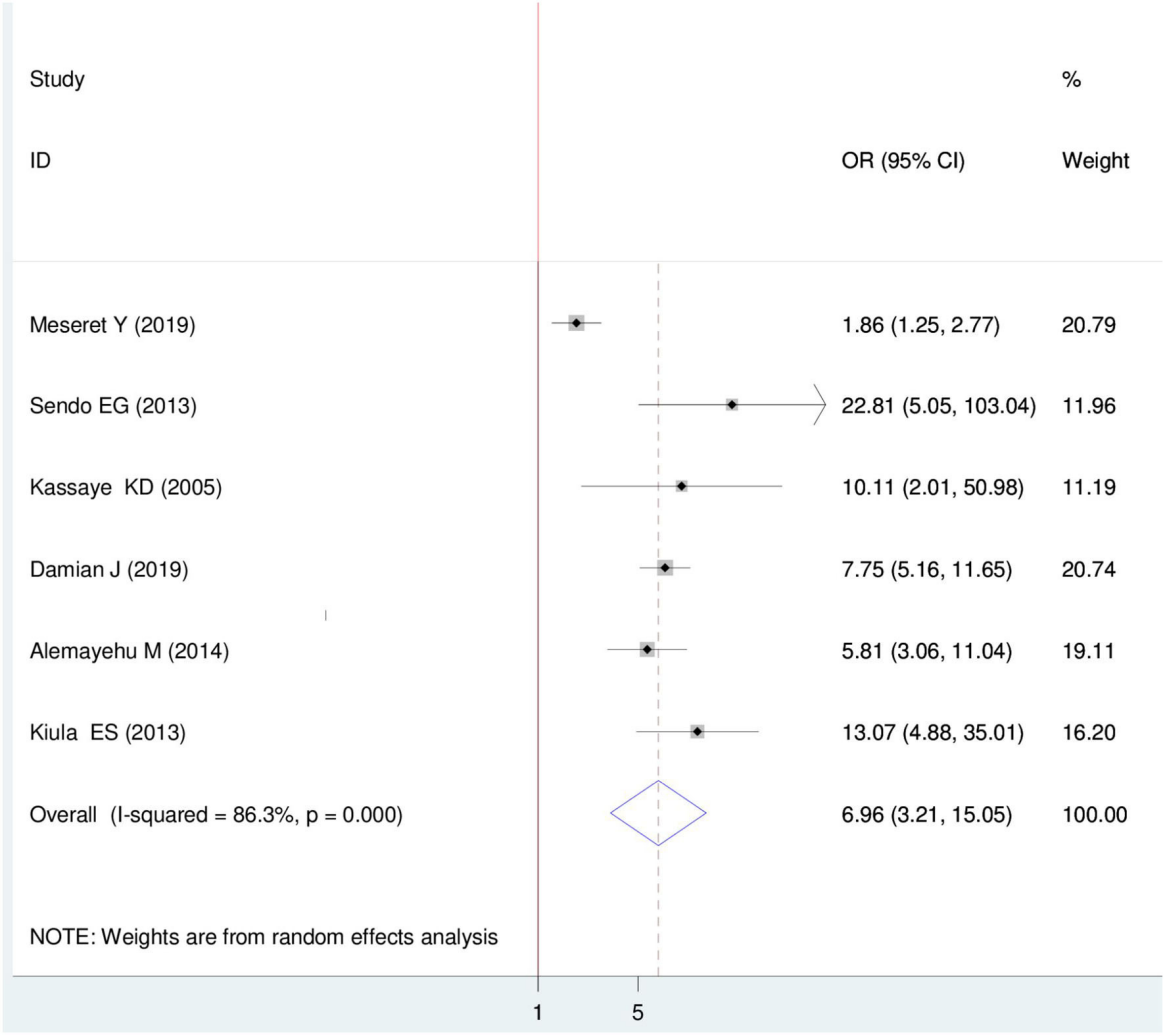
Forest plot of association between HIV sero-status disclosure and discussion on HIV before test among women in East Africa, 2021.

Six primary articles were included (22–24, 26, 33, 35). The pooled finding from this research showed that there is a relationship between discussing before the test and HIV sero-status disclosure (OR = 6.96 (95%, CI: 3.21, 15.05). The odds of disclosing HIV sero-status were 6.96 times higher among women who had a discussion on HIV before the test than their counterparts. For this Meta-regression, a random effect model was used (I^2^ = 86.3%, *P* = < 0.001) (Figure 9).

## Discussion

Despite major interventions that have been made to confront HIV epidemics; it remains a major public health problem globally. Disclosure of HIV positive among WLHIV is fundamental in PMTCT and to reduce HIV spreads within the community. Literature from East Africa reports the prevalence and factors associated with HIV sero-status disclosure differently and there is no pooled evidence regarding this issue. This systematic review and meta-analysis aimed to assess the pooled prevalence of HIV sero- positive disclosure status among women in East Africa. Accordingly, the pooled magnitude of HIV sero-positive disclosure among women in East Africa was 73.77% (95% CI: 67.76, 79.77). This is similar to the meta-analysis result conducted in Ethiopia (73%) (36) and South Africa (74.4%) (37), but lower than the study from Nigeria (88%) (38), Northern Nigeria (89%) (39), Enugu, Nigeria (96.7%) (40) and Southeastern Nigeria (97.1%) (41). Conversely, the current review finding was higher than the study conducted in sub-Saharan Africa (67%) (42) and Barbados (28.8%) (43). The possible reason for the variation might be the socio-economic, cultural, access to health care services, and year difference in primary studies.

Several factors contributed to the disclosure status of Women Living with HIV. Likewise, knowing the partner(s) sero-status, marital status, relationship before the test, and having a discussion on HIV with the sexual partner before the test were among identified factors. Those women who know their partners' HIV status were 10 times more likely to disclose their HIV sero-status. This is supported by a study from sub-Saharan Africa (42), and South Africa (44). This is probably because women who have HIV-positive partner can easily disclose their HIV sero-status as HIV-positive individual prefers to disclose to a partner with a known HIV-positive than to a negative or unknown HIV sero-status partner. Women who know their partner's status may have a discussion about HIV testing; this could facilitate the disclosure process. Furthermore, married women were more than two times more likely to disclose their HIV sero-status than those who are single. This is supported by study findings in Ogun Nigeria (45). Married couples have known each other and have common things together which may ease the disclosure process.

Additionally, having a smooth relationship with a partner increases HIV sero-status disclosure. A study from sub-Saharan Africa (42), and urban Nigeria (38) support this finding. The possible explanation; a smooth relationship between the couples encourages discussion on HIV testing and that might simplify the disclosure course. Moreover, women who have a discussion about HIV with their partners were seven times more likely to disclose their HIV sero-status than those who had no discussion. This finding is supported by previous meta-analyses (36). Discussion leads to agreement and trustworthiness. Couples who discussed HIV tests may also discuss the way to disclose their status.

HIV sero-status disclosure following voluntary counseling and testing has a great contribution in preventing the onward HIV infection community transmission; particularly averting MTCT. Therefore, the current review has an implication for policymakers and health care providers to further strengthen counseling and guiding couples, particularly women on further HIV sero-status disclosure.

## Strengths and limitations of the study

Even though various databases were extensively searched for both published articles, but not without limitations. The majority of the primary studies included in this review were cross-sectional in design; therefore, it is not possible to conclude the temporal relationship between the disclosure status and associated factors. As well, only articles published in the English language were included. Finally, some variables related to disclosure were not included in this review as we analyzed variables shown in the primary studies.

## Conclusion

Significant numbers of women are not disclosed their HIV sero-status. Knowing the partner's HIV sero-status, being married, having a smooth relationship, and discussion on HIV before the test were identified factors of disclosure status among WLHIV in East Africa. Therefore, policymakers and health care providers need to strengthen further disclosure of HIV sero-status among women.

## Data availability statement

The original contributions presented in the study are included in the article/supplementary material, further inquiries can be directed to the corresponding author/s.

## Author contributions

GM conceptualized the topic of the study. DB, DM, and BW searched and extracted articles. GM, EM, AT, and LB analyzed the data. AO, DC, and GF participated in data extraction, analysis, and write-up of the manuscript. All authors read and approved the final manuscript.

## Conflict of interest

The authors declare that the research was conducted in the absence of any commercial or financial relationships that could be construed as a potential conflict of interest.

The reviewer ET declared a shared affiliation with the authors to the handling editor at time of review.

## Publisher's note

All claims expressed in this article are solely those of the authors and do not necessarily represent those of their affiliated organizations, or those of the publisher, the editors and the reviewers. Any product that may be evaluated in this article, or claim that may be made by its manufacturer, is not guaranteed or endorsed by the publisher.

## Abbreviations

HIV: Human Immunodeficiency Virus
PMTCT: Preventing mother-to-child transmission
AOR: Adjusted Odds ratio
CI: Confidence Interval
JBI: Joanna Briggs Institute
WHO: World Health Organization
PRISMA: Preferred Reporting Items for Systematic Reviews and Meta-Analyses
WLHIV: Women Living with HIV.
